# Proteasome Inhibitors Decrease the Viability of Pulmonary Arterial Smooth Muscle Cells by Restoring Mitofusin-2 Expression under Hypoxic Conditions

**DOI:** 10.3390/biomedicines10040873

**Published:** 2022-04-09

**Authors:** I-Chen Chen, Yi-Ching Liu, Yen-Hsien Wu, Shih-Hsing Lo, Shu-Chi Wang, Chia-Yang Li, Zen-Kong Dai, Jong-Hau Hsu, Chung-Yu Yeh, Yu-Hsin Tseng

**Affiliations:** 1 Department of Pediatrics, Kaohsiung Medical University Hospital, Kaohsiung Medical University, Kaohsiung 80756, Taiwan; yljane.chen@gmail.com (I.-C.C.); furtherchia@gmail.com (Y.-C.L.); eddiewu1986@gmail.com (Y.-H.W.); allenjay66@gmail.com (S.-H.L.); zenkong@gmail.com (Z.-K.D.); jhh936@yahoo.com.tw (J.-H.H.); oeslees@gmail.com (C.-Y.Y.); 2 Department of Pediatrics, School of Medicine, College of Medicine, Kaohsiung Medical University, Kaohsiung 80708, Taiwan; 3 Graduate Institute of Medicine, College of Medicine, Kaohsiung Medical University, Kaohsiung 80708, Taiwan; chiayangli@kmu.edu.tw; 4 Department of Medical Laboratory Science and Biotechnology, Kaohsiung Medical University, Kaohsiung 80708, Taiwan; shuchiwang@kmu.edu.tw

**Keywords:** proteasome inhibitor, mitofusin-2, hypoxia, pulmonary hypertension

## Abstract

Pulmonary hypertension (PH) is a severe progressive disease, and the uncontrolled proliferation of pulmonary artery smooth muscle cells (PASMCs) is one of the main causes. Mitofusin-2 (MFN2) profoundly inhibits cell growth and proliferation in a variety of tumor cell lines and rat vascular smooth muscle cells. Down-regulation of MFN2 is known to contribute to PH. Proteasome inhibitors have been shown to inhibit the proliferation of PASMCs; however, there is no study on the regulation of proteasome inhibitors through MFN-2 in the proliferation of PASMCs, a main pathophysiology of PH. In this study, PASMCs were exposed to hypoxic conditions and the expression of MFN2 and cleaved-PARP1 were detected by Western blotting. The effects of hypoxia and proteasome inhibitors on the cell viability of PASMC cells were detected by CCK8 assay. The results indicated that hypoxia increases the viability and reduces the expression of MFN2 in a PASMCs model. MFN2 overexpression inhibits the hypoxia-induced proliferation of PASMCs. In addition, proteasome inhibitors, bortezomib and marizomib, restored the decreased expression of MFN2 under hypoxic conditions, inhibited hypoxia-induced proliferation and induced the expression of cleaved-PARP1. These results suggest that bortezomib and marizomib have the potential to improve the hypoxia-induced proliferation of PASMCs by restoring MFN2 expression.

## 1. Introduction

Pulmonary hypertension (PH) is a severe progressive disorder characterized by endothelial dysfunction and the uncontrolled proliferation of pulmonary artery smooth muscle cells and fibroblasts, resulting in increased pulmonary vascular resistance (PVR) and pulmonary pressures. In addition, fixed vascular obstruction with loss of the cross-sectional area is the major cause of increased PVR in most patients. This leads to reduced cardiac output, right heart failure, and premature death [1,2]. PH usually manifests in the 30- to 40-year-old age group, but it can also be diagnosed in children, in whom the prognosis is more severe [3], and can even be fatal in the absence of contemporary ameliorative treatment [4]. Current PH treatments mainly use vasodilators, which cannot directly solve vascular obstruction, nor can most therapies target the hypertrophied right ventricle (RV), which is fibrotic and ischemic due, in part, to microvascular rarefaction [5,6]. In fact, only 12.6% of patients respond to effective vasodilators. Despite several expensive treatments having already been approved, the mortality rate of PH is still very high [7,8], so a new treatment paradigm in PH is crucially needed to address the fundamental abnormalities caused by RV and pulmonary vasculature dysfunction.

Although abnormalities of platelets, endothelial cells, fibroblasts, and inflammatory cells play a key role in the pathogenesis of PH, the proliferation–apoptosis imbalance in pulmonary arterial smooth muscle cells (PASMCs) is one of the major causes of obstructive pulmonary vasculopathy. It has been reported that the mitochondrial network is fragmented in PH, and this destruction mechanism is related to the proliferation–apoptosis imbalance [9]. Mitochondria exist in a dynamic network comprised of rapid division (fission), continuous joining (fusion), and movement around the cell (trafficking) [10]. Mitochondria fusion and fission processes are both catalyzed by four dynamin-related GTPases in mammals, including dynamin-related protein 1 (DRP-1), mitofusin-1 (MFN1), mitofusin-2 (MFN2), and optic atrophy type 1 (OPA1) [10]. It is worth noting that *MFN2* was initially referred to as a hyperplasia suppressor gene when it was cloned because of its anti-proliferative effect [11,12], and has been reported to regulate cell proliferation and the apoptosis of multiple tumor cell lines and vascular smooth muscle cells [13,14]. In addition, the down-regulation of MFN2 contributes to various vascular proliferative disorders, including PH [11,15,16,17].

The ubiquitin–proteasome system (UPS) plays an essential role in the non-lysosomal degradation of various proteins, including those that require timely degradation for precise cell cycle regulation. Each β-ring of mammalian proteasomes contains three catalytic β-subunits (β1, β2, and β5), which are associated with caspase-like (CP-L), trypsin-like (T-L), and chymotrypsin-like (CT-L) activities, respectively [18]. The elevated function of UPS is found to be associated with the development of various diseases, such as cancer, and cardiovascular and neurological disorders [19,20]. Recent studies have indicated that proteasome inhibitors can inhibit the proliferation of vascular smooth muscle cells and restore endothelial function [21,22,23]; therefore, the inhibition of UPS by proteasome inhibitors is considered to be a novel therapeutic strategy for PH.

Many proteasome inhibitors have been successfully used in multiple myeloma treatment. Bortezomib is the first FDA-approved proteasome inhibitor for the treatment of multiple myeloma [20], and binds reversibly to the β5 ((CT-L)) subunit [18]. Marizomib is a newer drug in development and acts as an irreversible proteasome inhibitor of the β5, β2, and β1 subunits of the proteasome [18]. Marizomib has a unique efficacy and safety profile, with little cross-resistance with other proteasome inhibitors [20]. At present, there are few studies on the mechanism of proteasome inhibitors in the treatment of PH. In this study, we investigated the potential of bortezomib and marizomib in treating PH by restoring hypoxia-reduced MFN2 expression in primary human pulmonary artery smooth muscle cells.

## 2. Materials and Methods

### 2.1. Cell Culture and Drug Treatment

Primary human pulmonary artery smooth muscle cells (HPASMCs) were obtained from ScienCell Research Laboratories (ScienCell Research Laboratories, Carlsbad, CA, USA) and were cultured in a smooth muscle cell medium (cat. #1101, ScienCell Research Laboratories, Carlsbad, CA, USA) supplemented with 2% fetal bovine serum, 1% smooth muscle cell growth supplement, and 1% penicillin–streptomycin in a humidified incubator at 37 °C containing 5% CO_2_. HPASMCs were passaged at 70% to 80% confluence by dissociation from plates with 0.25% trypsin–EDTA and passages 5 to 12 were used in the experiment. When performing hypoxia experiments, HPASMCs were incubated under 1% Oxygen. The concentration of oxygen was controlled by an oxygen controller (model ProOx 110, BioSpherix Ltd., Redfield, NY, USA), which senses oxygen inside the chamber and infuses either nitrogen to reduce the concentration or oxygen to raise the concentration. Cobalt chloride (CoCl_2_), bortezomib, and marizomib were purchased from Sigma-Aldrich Inc. (St. Louis, MO, USA).

### 2.2. Cell Viability Assay

Cell viability in cell proliferation and cytotoxicity assays was measured using the cell counting kit-8 (CCK-8) assay kit (Sigma Aldrich Inc., MO, USA). HPASMCs were seeded at a density of 5000 cells per well in 96-well tissue culture plates and incubated at 37 °C and 5% CO_2_. Hypoxia experiments, MFN2 transfection, and proteasome inhibitors (bortezomib and marizomib) treatment were performed following overnight adherence. Then, 10 μL CCK-8 solution was added to each well and the plate was incubated at 37 °C for 2 h. Specific and reference absorbance levels were measured at 450 and 650 nm, respectively, on an enzyme-linked immunosorbent assay reader.

### 2.3. Western Blotting

Cells were gently washed twice by cold 1x PBS, and lysed using a RIPA buffer (25 mM Tris HCl, pH 7.6, 150 mM NaCl, 1% NP-40, 1% sodium deoxycholate, 0.1% sodium dodecyl sulfate; Thermo Fisher Scientific, Waltham, MA, USA) with a protease inhibitor cocktail reagent (Thermo Fisher Scientific, Waltham, MA, USA). The lysed cells were centrifuged at 13,000× *g* for 15 min at 4 °C, then, supernatant proteins were collected for Western blotting. Protein concentrations were measured using a Pierce BCA protein assay kit (Thermo Fisher Scientific, Waltham, MA, USA). Protein lysates (20 µg) were loaded on Blot 4–12% Bis-Tris Plus gels (Invitrogen, Carlsbad, CA, USA) and transferred to polyvinylidene difluoride membranes. Membranes were incubated in a blocking buffer for 1 h at room temperature. The membranes were incubated overnight with primary antibodies in PBST against human HIF-1α (cat# 36169, 1:1000, Cell Signaling Technology, Danvers, MA, USA), MFN2 (cat# 9482, 1:1000, Cell Signaling Technology, Danvers, MA, USA), pro-PARP1 (cat# ab32138, 1:1000, Abcam, UK), and cleaved-PARP1 (cat# ab32064, 1:15,000, Abcam, UK), Actin (cat# NB600-501, 1:30,000, Novus Biologicals, Littleton, CO, USA) at 4 °C. After washing off the primary antibodies, the blots were incubated with horseradish peroxidase-linked secondary antibodies (cat# NA9310 or cat# NA9340, 1:10,000, Cytiva, USA) for 1 h at room temperature, and the reading was performed using an enhanced chemiluminescence assay kit (Thermo Fisher Scientific, Waltham, MA, USA).

### 2.4. MFN2 Overexpression

To overexpress MFN2, one to ten micrograms of MFN2/pCMV3 were transfected into HPSMCs using Lipofectamine 3000 (Thermo Fisher Scientific, Waltham, MA, USA), and further cultured in a smooth muscle cell medium with 2% fetal bovine serum, 1% smooth muscle cell growth supplement, and 1% penicillin–streptomycin for subsequent experiments.

### 2.5. Statistical Analysis

Data were analyzed by GraphPad Prism5.0 (GraphPad Software, Inc., San Diego, CA, USA). An unpaired *t*-test was used to compare the mean between two independent groups. The one-way ANOVA was used to compare the means of more than two groups, and the Newman–Keuls method was used as a post hoc test following ANOVA. Data are shown as means ± standard error of three independent experiments. Statistical significance was set at *p* < 0.05.

## 3. Results

### 3.1. The Role of Mitofusin-2 (MFN2) in the Hypoxia-Induced Proliferation of Human Pulmonary Artery Smooth Muscle Cells

**Figure 1:**
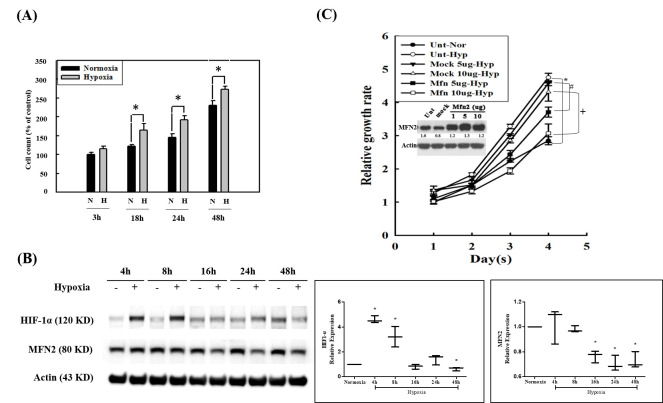
The role of MFN2 in hypoxia-induced proliferation. (**A**) HPASMCs were exposed to hypoxic (1% oxygen) or normoxic conditions for 3, 18, 24, and 48 h, respectively. Cell viability was detected using a CCK8 assay. * *p* < 0.05 hypoxic group versus normoxic group. The statistical difference was analyzed by an unpaired *t*-test and the data represented by means ±standard error of three independent experiments. (**B**) HPASMCs were exposed to the hypoxic (+) or normoxic (−) conditions for different times. Expression of HIF-1α and MFN2 was detected by Western blotting. Expression of actin was used as a loading control. The boxplots on the right show HIF-1 α/actin and MFN2/actin ratios under hypoxia conditions divided by HIF-1 α/actin and MFN2/actin ratios under normoxic conditions, respectively. The statistical analysis was performed with the normoxic conditions at each time point as 1. * *p* < 0.05 hypoxic group versus normoxic group. The statistical difference was analyzed by an unpaired *t*-test and the data represented by means ±standard error of three independent experiments. (**C**) HPASMCs were transiently transfected with the vector alone or the MFN2 plasmid. The expression of MFN2 was determined by Western blotting. Data indicated that the transfection condition is appropriate to successfully promote the expression of MFN2 in PASMCs. One, five, and ten micrograms of MFN2/pCMV3 transfection can effectively increase the expression of MFN2 in HPSMCs. Cell viability of five and ten micrograms of MFN2/pCMV3 transfected groups was detected at 1 to 4 days using a CCK8 assay. Both five and ten micrograms of the MFN2/pCMV- transfected groups inhibited the hypoxia-induced proliferation of HPASMCs. The statistical difference was analyzed by an unpaired *t*-test and the data are represented by means ±standard error of three independent experiments. * *p* < 0.05 hypoxic group (○) versus normoxic group (●). ^#^ *p* < 0.05, 5 μg MFN2 (■) versus 5 μg mock (▼). ^+^ *p* < 0.05, 10 μg MFN2 (□) versus 10 μg mock (Δ).

To examine the growth of human pulmonary artery smooth muscle cells (HPASMCs) under hypoxic or normoxic conditions, cells were maintained in hypoxic or normoxic condictions for 3, 18, 24 m and 48 h. Cell viability was detected using the CCK-8 assay. The results showed that cell viability was higher under hypoxic conditions (1% oxygen) for 18, 24, or 48 h than that of cells under normoxic conditions (Figure 1A). We further examined the protein expression of HIF-1α and MFN2 using Western blotting. The results showed that the expression of MFN2 decreased with the increase in hypoxia time. However, the expression of HIF-1α increased in the early stage of hypoxia, and gradually recovered with the prolongation of hypoxia time, and the expression of HIF-1α decreased under hypoxic conditions for 48 h (Figure 1B). To further examine the role of MFN2 in modulating the proliferation of HPASMCs under hypoxic conditions, HPASMCs were transiently transfected with plasmids directing the expression of MFN2, then cell viability was detected at 1 to 4 days. The results showed that cell viability under hypoxic conditions was higher than that under normoxic conditions, and the overexpression of MFN2 inhibited the proliferation of HPASMCs induced by hypoxia (Figure 1C).

### 3.2. The Effect of Proteasome Inhibitors in the Hypoxia-Inhibited MFN2 Expression and Hypoxia-Inhibited Apoptosis

**Figure 2:**
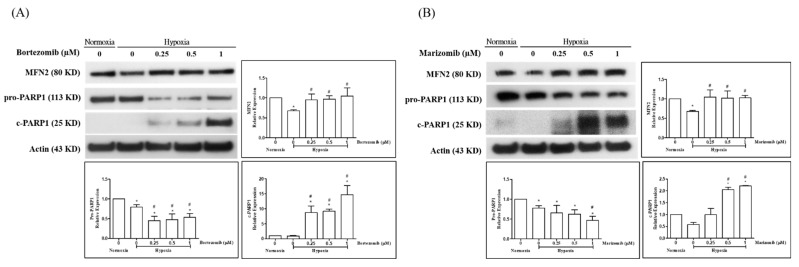
Proteasome inhibitors restored the expression of MFN2 and induced apoptosis under hypoxic conditions. HPASMCs were exposed to hypoxic conditions and were treated with bortezomib and marizomib for 24 h. The expression of MFN2, pro-PARP1, and cleaved PARP1 were detected by Western blotting. The expression of actin was used as a loading control. Bortezomib (**A**) and marizomib (**B**) restored the expression of MFN2-inhibited by hypoxia, inhibited the levels of pro-PARP1, and induced the level of cleaved PARP1. One-way ANOVA was used to determine the differences between the experimental and vehicle control (DMSO) groups and the Newman–Keuls Multiple Comparison Test was used as a post hoc test following ANOVA. The data represent means ±standard error of three independent experiments. * *p* < 0.05 versus normoxic group. # *p* < 0.05 versus hypoxic control group.

To examine the effect of proteasome inhibitors on the MFN2 expression of HPASMCs under hypoxia conditions, HPASMCs were exposed to hypoxic conditions and were treated with bortezomib and marizomib for 24 h. Expression of MFN2, pro-PARP1, and cleaved-PARP1 were detected by Western blotting. Actin was used as a loading control. Poly (ADP-ribose) polymerase-1 (PARP-1) is one of several well-known cellular substrates of caspases. PARP1 was cleaved into fragments by caspases during apoptosis; thus, cleaved PARP1 is a useful marker of apoptosis [24,25]. The results showed that hypoxia inhibited the expression of MFN2. Bortezomib (Figure 2A) and marizomib (Figure 2B) could restore hypoxia-inhibited MFN2 and increase cleaved PARP1 expression.

### 3.3. The Effects on Cell Viability and Apoptosis of Bortezomib and Marizomib in HPASMCs under Hypoxic and Normoxic Conditions

**Figure 3:**
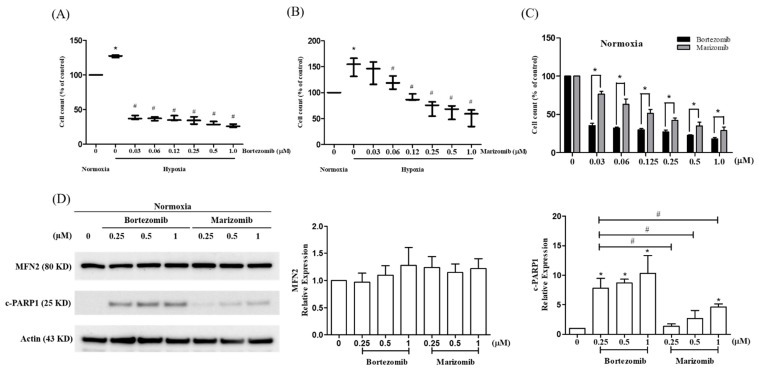
Proteasome inhibitors inhibit hypoxia-induced cell proliferation and marizomib has less cytotoxicity than bortezomib in HPASMCs. The cell viability was detected by a CCK-8 assay and expressions of MFN2 and cleaved PARP1 were detected by Western blotting. Hypoxia induced cell proliferation while, bortezomib (**A**) and marizomib (**B**) treatments for 24 h could inhibit hypoxia-induced proliferation under hypoxic conditions. One-way ANOVA was used to determine the differences between experimental and vehicle control (DMSO) groups and the Newman–Keuls Multiple Comparison Test was used as a post hoc test following ANOVA. Data are represented by means ±standard error of three independent experiments. * *p* < 0.05 versus the vehicle control group (DMSO) under normoxic conditions. # *p* < 0.05 versus the vehicle control group (DMSO) under hypoxic conditions. (**C**) Different doses of bortezomib and marizomib treatments for 24 h under normoxic conditions. The cytotoxicity of marizomib was lower than that of bortezomib. An unpaired *t*-test was used to analyze the differences between bortezomib and marizomib treatments. Data are represented by means ±standard error of three independent experiments. * *p* < 0.05 versus bortezomib treatment. (**D**) Under normoxic conditions, bortezomib treatment for 24 h increased higher levels of cleaved PARP1 than marizomib treatment. Bortezomib and marizomib treatments did not affect the expression of MFN2 under normoxic conditions. A one-way ANOVA was used to determine the differences between the experimental and vehicle control (DMSO) groups and the Newman–Keuls Multiple Comparison Test was used as a post hoc test following ANOVA. Data are represented by means ±standard error of three independent experiments. * *p* < 0.05 versus the vehicle control group (DMSO). # *p* < 0.05 versus bortezomib treatment at the same concentrations.

To further validate the effect of proteasome inhibitors on the cell viability of HPASMCs under normoxic or hypoxic conditions, cells were exposed to normoxic or hypoxic conditions and synchronously treated with bortezomib and marizomib for 24 h. The cell viability was detected by a CCK-8 assay, and the expression of apoptosis-related proteins was detected by Western blotting. The results showed that hypoxia induced cell proliferation, and bortezomib (Figure 3A) and marizomib (Figure 3B) inhibited hypoxia-induced proliferation. Under normoxic conditions, the cytotoxicity of marizomib was lower than that of bortezomib in HPASMC cells (Figure 3C). In addition, bortezomib treatment increased higher levels of cleaved PARP1 than marizomib treatment under the same drug concentration (Figure 3D). However, bortezomib and marizomib treatments did not affect the expression of MFN2 under normoxic conditions.

### 3.4. The Effect of Proteasome Inhibitors in a Chemical Hypoxia Model

**Figure 4:**
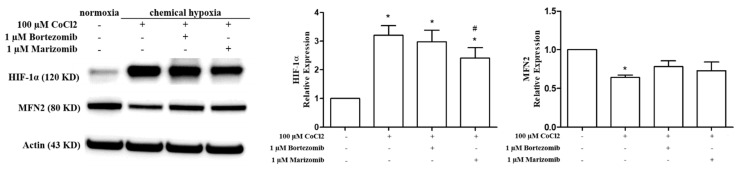
Proteasome inhibitors cannot obviously restore the expression of MFN2 inhibited by chemical hypoxia. HPASMCs were pretreated with 100 µM CoCl_2_ for 2 h, followed by bortezomib or marizomib for 24 h. The expressions of HIF-1α and MFN2 were detected by Western blotting. Equal loading was confirmed with an anti-actin antibody. The expressions of HIF-1α in the CoCl_2_ alone group, the CoCl_2_ plus bortezomib group, and the CoCl_2_ plus marizomib group were higher than those in the normoxia group, and the expression of MFN2 was lower than that in the normoxia group. A one-way ANOVA was used to determine the differences between the experimental and control groups, and the Newman–Keuls Multiple Comparison Test was used as a post hoc test following the ANOVA. Data are represented by means ±standard error of three independent experiments. * *p* < 0.05 vs. normoxia group. # *p* <0.05 versus the CoCl_2_ alone group.

**Figure 5:**
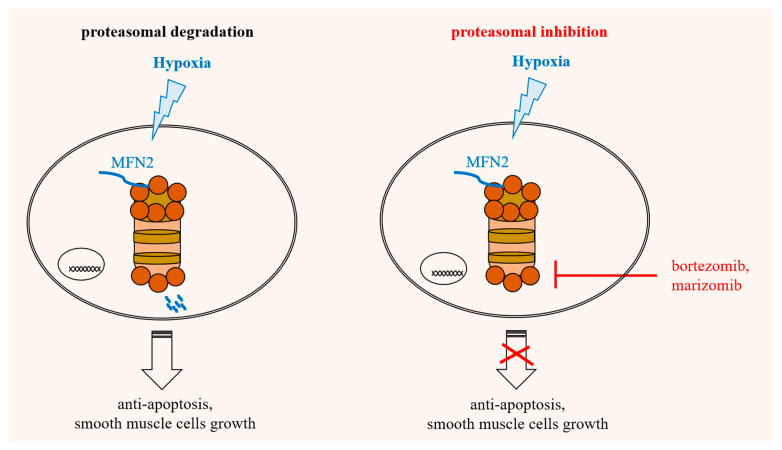
Schematic diagram of proteasome inhibitors restoring the physical hypoxia-inhibited MFN2 expression, inducing apoptosis, and inhibiting the hypoxia- induced proliferation in HPASMCs. The left image shows that hypoxia increases the proteasomal degradation of MFN2, inhibiting apoptosis and promoting cell growth. The right image shows that bortezomib and marizomib blocked the activity of the proteasome, inhibiting the ability of anti-apoptosis and cell growth.

Cobalt chloride (CoCl_2_) treatment is another commonly used chemical hypoxia model [26]. HPASMCs were pretreated with 100 µM CoCl_2_ (cobalt chloride) for 2 h, followed by bortezomib or marizomib for 24 h. The expressions of HIF-1α and MFN2 were detected by Western blotting. The results showed that CoCl_2_ induced the expression of HIF-1α and inhibited the expression of MFN2; however, 1 µM bortezomib or marizomib did not obviously restore the expression of MFN2 inhibited by chemical hypoxia (Figure 4). Furthermore, a schematic diagram of the restoration of the physical hypoxia-inhibited MFN2 expression, the induction of apoptosis, and the inhibition of hypoxia-induced proliferation by proteasome inhibitors in HPASMCs is shown in Figure 5.

## 4. Discussion

Studies have found similarities between PH and cancers, such as the expression of malignant cell biomarkers and the resistance to programmed cell death [27]. Based on anti-tumor activity in a wide range of preclinical cancer models, targeting the UPS has emerged as a rational apoptosis-based approach to reverse pulmonary vascular remodeling in PH. Although the potential toxicity of these inhibitors remains a concern, proteasome inhibitors, including bortezomib and carfilzomib, have been reported to have beneficial effects in reducing pulmonary artery wall thickness [27]. In addition, the down-regulation of MFN2 is known to contribute to PH, and MFN2 is considered to be a novel therapeutic target for the treatment of PH. In the present study, the effect of proteasome inhibitors on MFN2 degradation under hypoxic conditions in HPASMCs was investigated. Our results indicated that proteasome inhibitors (bortezomib and marizomib) could restore the expression of MFN2-inhibited by hypoxia, inhibit hypoxia-induced proliferation, and induce cell apoptosis in HPASMCs.

Hypoxia-inducible factor-1 (HIF-1) is a major regulator of oxygen homeostasis and is involved in gene transcription including angiogenesis, vascular reactivity and remodeling, vasomotor control, glucose and energy metabolism, erythropoiesis, and cell proliferation and viability [28]. HIF-1 is composed of the subunits HIF-1α and HIF-1β, of which HIF-1α has been described as an endogenous hypoxic marker [29]. Previous studies have shown that the right ventricular systolic pressure (RVSP) in a HIF-1α knockout animal model (*Hif1α* *^+/–^* mice) exposed to hypoxia was significantly lower than that in the hypoxic control group [30,31,32]. These results demonstrate that HIF-1α in the pulmonary arterial smooth muscle cells contributes to an increased RVSP and chronic hypoxia-induced pulmonary vascular remodeling. In addition, the ability of HIF-1α to regulate the expression of MFN2 has been reported. HIF-1α affects the differentiation of the neural stem cell (NSC) from human-induced pluripotent stem cells (hiPSCs) through MFN2-mediated Wnt/β-catenin signaling. HIF-1α knockdown significantly enhances the expression of MFN2, and in turn, MFN2 can reverse the effects of NSC differentiation caused by HIF-1α [33]. In addition, the ubiquitin–proteasome system is involved in the regulation pathway of HIF-1α. Proteasomal inhibition has been shown to stabilize the HIF-1a protein and induce the formation of the HIF-1 complex in a manner similar to hypoxia stimulation [34,35]. Our results showed that hypoxia induced the expression of HIF-1α and inhibited the expression of MFN2 in HPSMCs. Proteasome inhibitors (bortezomib and marizomib) can restore hypoxia-inhibited MFN2 expression. These results indicated that proteasome inhibitors could reverse the effects of HIF-1α overexpression by restoring MFN2 expression in HPSMCs under hypoxic conditions.

Bortezomib, the first FDA-approved proteasome inhibitor for the treatment of multiple myeloma, is known to attenuate PH in hypoxia- and monocrotaline-induced PH models by inhibiting the proliferation of vascular smooth muscle cells and ameliorating endothelial dysfunction [22]. Our study is the first to indicate that bortezomib ameliorates the hypoxia-induced proliferation of PASMCs by restoring MFN2 expression. Despite the apparent success of bortezomib in the treatment of multiple myeloma, a subset of patients fail to respond to bortezomib therapy or develop resistance when they relapse [36]. A next-generation proteasome inhibitor has been developed to improve disease cure rates. For example, carfilzomib, a next-generation FDA-approved proteasome inhibitor, exerts its inhibitory function by binding to the β5 (CT-L activity) subunit. However, unlike bortezomib, the binding of carfilzomib to the β5 subunit is irreversible [37]. Carfilzomib shows a lower recovery of proteasome inhibition compared to bortezomib over the 24 h culture period in parental and bortezomib-resistant cells; that is, carfilzomib maintains its cytotoxic potential in the bortezomib-resistant cell lines [36]. It is speculated that resistance to bortezomib can be overcome by irreversible inhibitors. In addition, although bortezomib induced a significant anti-tumor activity in pre-clinical models [38,39], it did not show efficacy in clinical trials in triple-negative breast cancer (TNBC), either alone or in combination with other therapies [38]. Bortezomib inhibits the CT-L activity without any significant effect on T-L and CP-L activity. Inhibition of CT-L by bortezomib causes the compensatory activation of T-L and CP-L, resulting in patient resistance to bortezomib [40]. The development of an irreversible pan-proteasome inhibitor might be an effective way to overcome drug resistance.

Marizomib is a newer drug in development and acts as an irreversible pan-proteasome inhibitor of the β5, β2, and β1 subunits [40]. Clinical trials have demonstrated that marizomib is well-tolerated, either as monotherapy or in combination with poma-lidomide and displays good activity in relapsed and refractory multiple myeloma [40,41]. In addition, marizomib induces better anti-tumor responses when used alone or in combination with standard-of-care chemotherapy in triple-negative breast cancer cell lines and patient-derived xenografts [40]. Marizomib-inhibited oxidative phosphorylation (OXPHOS) upregulates glycolysis to meet the energetic demands of TNBC cells and the combined inhibition of glycolysis with marizomib results in a synergistic anti-cancer activity, which indicates that marizomib is a dual inhibitor of proteasome and oxidative phosphorylation (OXPHOS) in triple-negative breast cancer [40]. Our results showed that although bortezomib and marizomib were similar in their ability to restore the expression of MFN2 in a hypoxia-induced proliferation model of HPASMCs (Figure 2), at the same concentration of bortezomib and marizomib, marizomib caused a lower decrease in cell viability than bortezomib under both normoxic and hypoxic conditions (Figure 3). It is reasonable to speculate that marizomib has the potential to be a clinical drug that could be the next generation proteasome inhibitor with a lower toxicity and with the ability to overcome drug resistance.

Previous studies have reported that hypoxia increased proteasome activity in isolated pulmonary artery tissues [42,43]. The expression of MFN2 decreased and the phosphorylation of MFN2 increased in PASMCs from patients with pulmonary arterial hypertension (PAH) [44]. The expression of MFN2 in PAH PASMC is known to be lower than that in normal PASMCs [17]. Elevating MFN2 inhibits the proliferation of PAH PASMCs, whereas reducing MFN2 induces the proliferation of normal PASMCs [16,17]. Chronic hypoxia is known to be a common cause of pulmonary hypertension, and hypoxia-induced effects can be reversed by the overexpression of MFN2 in PASMCs. In addition, MFN2 is down-regulated by proteasomal degradation triggered by PINK1, which phosphorylates MFN2 at S422 [17]. MG-132, which inhibits proteasome function, is a proteasome inhibitor and has been shown to increase the steady-state levels of MFN2 [44]. MG-132 is used in laboratories but has not yet received FDA approval. Bortezomib is the first proteasome inhibitor to receive FDA approval for the treatment of multiple myeloma in 2003 [45,46], and marizomib is a second-generation proteasome inhibitor in clinical trials [18]. In this study, we investigated whether bortezomib and marizomib could avoid the downregulation of MFN2 under hypoxia conditions and whether bortezomib and marizomib inhibited the hypoxia-induced cell viability of PASMCs by restoring the expression of MFN2. The results showed that bortezomib and marizomib have the potential to improve the hypoxia-increased cell viability of PASMCs by restoring MFN2 expression but did not affect the expression of MFN2 under normoxic conditions.

In our study, both chemical hypoxia (CoCl_2_) and physical hypoxia (1% O_2_) models were used to investigate whether proteasome inhibitors could restore the expression of MFN2 inhibited by hypoxia. Our results showed that both bortezomib and marizomib could restore the expression of MFN2 inhibited under physical hypoxia (Figure 2) but could not restore the expression of MFN2 inhibited under chemical hypoxia (Figure 4). In addition, marizomib cannot stabilize the expression of HIF-1α under chemical hypoxia (Figure 4). A previous study indicated that the hypoxic effects induced by chemical and physical hypoxia are not the same in cardiomyocytes, and the physical hypoxia model is considered to mimic the actual physiological hypoxia situation [47]. Therefore, even though the chemical hypoxia model is easier to operate than the physical hypoxia model, the chemical hypoxia model might not reflect real physiological conditions. For the investigation of hypoxia-related mechanisms, the choice of hypoxia model needs to be carefully considered.

Several studies have reported that hypoxia leads to an increase in the production of superoxide anion, the main reactive-oxygen-species (ROS), in PASMCs [48,49]. MFN2-silencing elevates mitochondrial ROS levels that in turn increased melanogenesis in a B16 mouse melanoma cell line-based low-density culturing-induced pigmentation model [50]. MFN2 deficiency impairs mitochondrial membrane potential and ROS production in the macrophage [51]. However, the association between superoxide anion production and MFN2 expression in PASMCs has not been investigated. Whether bortezomib and marizomib can suppress superoxide anion production by inducing the expression of MFN2 in PASMCs is worth investigating in future.

In addition, the limitation of this study is that the activity of proteasome inhibitors does not target a single protein, so more completely activated signaling pathways under MFN2 are worthy of further investigation. Nevertheless, bortezomib and marizomib decrease the viability of PASMCs by restoring the expression of MFN2 under hypoxic conditions; these findings provide novel therapeutic strategies for pulmonary hypertension.

## 5. Conclusions

Bortezomib is known to attenuate PH in hypoxia- and monocrotaline-induced PH models by inhibiting the proliferation of vascular smooth muscle cells and improving endothelial dysfunction. Our study is the first to indicate that bortezomib ameliorates the hypoxia-induced proliferation of HPASMCs by restoring the expression of MFN2. We also found that the cytotoxicity of marizomib is lower than that of bortezomib. Although marizomib is a newer drug in development, it has the potential to be the next generation proteasome inhibitor with a lower toxicity and an ability to overcome drug resistance.

## Data Availability

Not applicable.
